# Chemical Devulcanization of Crosslinked Nitrile Rubber Using Tetra-*n*-Butylammonium Fluoride (TBAF) as a Devulcanization Aid

**DOI:** 10.3390/ma19153317

**Published:** 2026-08-04

**Authors:** Jakub Wręczycki, Katsiaryna Nauharodskaya, Dariusz M. Bieliński, Grzegorz Mlostoń

**Affiliations:** 1 Institute of Polymer and Dye Technology, Faculty of Chemistry, Lodz University of Technology, 16 Stefanowskiego Street, 90-537 Lodz, Poland; 240350@edu.p.lodz.pl (K.N.); dariusz.bielinski@p.lodz.pl (D.M.B.); 2 Department of Organic and Applied Chemistry, Faculty of Chemistry, University of Lodz, 12 Tamka Street, 91-403 Lodz, Poland; grzegorz.mloston@chemia.uni.lodz.pl

**Keywords:** rubber, vulcanizates, crosslinks, devulcanization, tetra-*n*-butylammonium fluoride

## Abstract

Utilization of tetra-*n*-butylammonium fluoride (TBAF) as a new, hitherto unknown devulcanization aid in the chemical devulcanization of sulfur crosslinked diene rubber has been demonstrated. The influences of crosslink structure (the ratio of mono-, di-, and polysulfidic bonds) and carbon black (CB) loading on the efficiency of acrylonitrile-butadiene-rubber (NBR) devulcanization have been studied. The rubber vulcanizates were treated with TBAF solutions under varying conditions (solution concentration, solvent type, temperature, and reaction time). Changes in crosslink density and other relevant network properties of the rubber vulcanizates were measured. Results showed a significant reduction in crosslink density—up to 50% after chemical treatment. The fluoride-based approach, in which fluoride salt is a source of nucleophilic fluoride anions (F^−^), can cleave the sulfidic crosslinks, demonstrating significant potential for efficient recycling of used rubber vulcanizates by their devulcanization.

## 1. Introduction

### 1.1. Network Structure of Crosslinked Rubber

From the perspective of elastomer technology, two opposing processes are fundamental: vulcanization (crosslinking) and devulcanization (de-crosslinking). While vulcanization transforms raw rubber into a unique material of high elasticity, suitable for industrial use, devulcanization enables the recovery of its strategic components, including raw rubber.

The crosslink structure of rubber is highly important because it directly determines its functional and engineering properties. Curing of rubber results in a three-dimensional architecture in which rubber macromolecules are combined through chemical bonds and physical interconnections (chemical and physical crosslinks, respectively). Hence, the rubber network can be described on two levels: microphysical (geometric arrangement) and molecular (chemical composition). The density and structure of crosslinks govern key characteristics such as elasticity, tensile strength, and deformation resistance. Understanding the network microstructure is essential for predicting and optimizing material performance and applications.

Chemical crosslinks typically consist of sulfidic bonds of different length or direct carbon–carbon bonds, and are formed through copolymerization or coupling of functionalized chain ends. However, no network is ideal, and various structural defects such as free chain ends and cyclic structures (loops) along macromolecules are also present, which contribute a little to the desirable elastic response of rubber vulcanizates [1,2,3,4,5,6].

At the molecular level, sulfur crosslinks are classified as monosulfidic (–C–S–C–), disulfidic (–C–S–S–C–), or polysulfidic (–C–S_x_–C–, where x ≥ 3). The resulting network composition depends on vulcanization parameters (temperature, pressure) and the curing system (sulfur content, accelerator type and concentration, activators, fillers and other additives). Conventional systems with high sulfur content favor polysulfidic crosslinks, while efficient systems with higher accelerator-to-sulfur ratios produce predominantly mono- and disulfidic bonds [4,7].

The crosslink network structure determines not only mechanical and chemical stability but also recyclability of rubber vulcanizates. Because sulfur-based crosslinks, particularly of the short type (monosulfidic), exhibit high resistance to thermal and chemical degradation, devulcanization, the reversal of vulcanization, remains a significant scientific and technological challenge.

### 1.2. Devulcanization

Devulcanization is the process of reversing the crosslinking state of an elastomer, involving the selective breaking of chemical bonds in the three-dimensional network of vulcanized rubber (e.g., sulfur bridges), while leaving the main polymer chain intact.

Partial/selective cleavage of crosslinks is used analytically to investigate network structure (for example, thiol-amine analysis [1,8]), while their complete cleavage is desirable for recycling vulcanized rubber, particularly coming from end-of-life tires or other rubber wastes. The recycling of used rubber products is currently one of the key ecological and environmental challenges. Its effective implementation helps reduce waste, recover key strategic raw materials (rubbers, including natural rubber, and fillers), and minimize the negative impact of rubber wastes on the environment. Due to that, various devulcanization methods have been explored, including mechanical, chemical, biological, ultrasonic, and microwave techniques [9,10,11,12,13].

Chemical devulcanization demonstrates a strong potential for selective bond cleavage. Common reagents applied in the crosslink structure studies (analytical approach) include thiol-amine systems (usually a combination of dodecane-1-thiol and propane-2-thiol with piperidine) [1,8], lithium aluminum hydride (LiAlH_4_) [14,15,16], or phosphines (e.g., triphenylphosphine; Ph_3_P) [17,18]. On the other hand, a broad group of chemicals that are reactive towards sulfidic bonds is used as supportive devulcanization aids in the thermomechanical treatment, realized in laboratory mixers or extruders [13]. This includes organic sulfides [13,19,20] (e.g., DPDS—diphenyl disulfide, TESPT—bis-3-triethoxy-silyl-propyltetra sulfide), blocked mercapto silanes [21] (e.g., trialkoxy-mercapto-alkylsilane), organic peroxides [22] (e.g., DCP—dicumyl peroxide, BPO—benzoyl peroxide), chemicals used as rubber additives [23] (e.g., accelerators: CBS—N-cyclohexylbenzothiazole-2-sulfenamide, antidegradants: 6PPD—*N*-(1,3-dimethylbutyl)-*N*’-phenyl-*p*-phenylenediamine). However, many of the effective reagents were tested decades ago and are currently environmentally problematic or toxic to human health, making their application concerning [9]. For example, diphenyl disulfide, during thermal processing, forms extremely toxic thiophenol (PhSH) [24,25].

Therefore, alternative devulcanization systems that promote selective cleavage under milder conditions are of high interest. Fluoride salts, being a source of fluoride anion, a nucleophilic species in nonaqueous media, represent one such alternative. Tetra-*n*-butylammonium fluoride (TBAF) is an interesting candidate for devulcanization studies due to its unique reactivity profile [26,27,28].

### 1.3. Tetrabutylammonium Fluoride (TBAF) and Its Potential Use in Elastomer Chemistry

**Figure 1:**
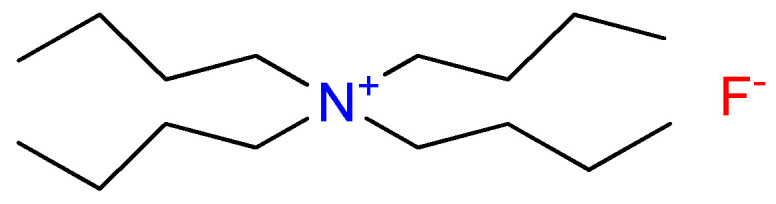
Chemical structure of Tetrabutylammonium fluoride (TBAF).

Tetrabutylammonium fluoride (TBAF), with molecular formula (C_4_H_9_)_4_N^+^F^−^ (Figure 1), is an organic quaternary ammonium salt. The hydrophobic TBA^+^ cation promotes solubility in organic solvents such as THF, dichloromethane, and acetonitrile. Its bulky structure limits effective solvation of the fluoride anion, rendering the fluoride relatively “naked” and more reactive than in inorganic salts with small, strongly coordinating cations (e.g., KF—potassium fluoride, NaF—sodium fluoride, CsF—cesium fluoride) [26,27,28]. Consequently, TBAF is a widely used source of reactive fluoride ions in nonaqueous media, for example, in silyl deprotection and as a phase-transfer catalyst [29,30,31], or in nucleophilic aromatic fluorination (SNAr) reactions [32,33]. Successful application of TBAF as a nucleophilic activator of elemental sulfur (S_8_) and furthermore a sulfur-transferring reagent through fluoropolysulfide anion (FS_x_^−^) to thioketones and thiiranes leading to a variety types of sulfur-rich heterocyclic compounds [34,35] and sulfur-containing polymers [36], respectively, has been described in our previous works.

Research on the use of fluoride salts in elastomer systems remains limited but is expanding. Notably, work by Wręczycki et al. demonstrated that adding fluoride salts to conventional sulfur–accelerator curing systems can significantly reduce the vulcanization temperature of diene rubbers (NBR, SBR, NR, BR, EPDM) up to 120 °C or shorten the vulcanization time at the traditional vulcanization temperature of 160 °C [37,38].

Herein we present our investigation of applying fluoride salt (TBAF) as a devulcanization aid in the direct chemical devulcanization process. The interaction of the fluoride anion with sulfide bonds in the crosslinks of the rubber network, as well as with model alkyl sulfides, is discussed.

## 2. Materials and Methods

### 2.1. Materials

Reagents and research materials with the purity and characteristics indicated below were obtained from the following suppliers: Sigma-Aldrich (Merck) (Darmstadt, Germany): elemental sulfur (99.98%) (S_8_), tetra-*n*-butylammonium fluoride trihydrate (>97%) (TBAF), piperidine (99%); Acros Organics (Geel, Belgium): propane-2-thiol (98%), dodecane-1-thiol (98%); POCH (Avantor) (Gliwice, Poland): toluene (99.5%); Eurochem BGD Ltd. (Tarnów, Poland): tetrahydrofuran (THF) (analytical grade); Deutero GmbH (Kastellaun, Germany): deuterated chloroform (CDCl_3_) (99.8%); Lanxess (Cologne, Germany): zinc oxide (ZnO), *N*-cyclohexyl-2-benzothiazolesulfenamide (CBS), stearic acid; Versalis S.p.A. (San Donato Milanese, Italy): butadiene-acrylonitrile rubber (NBR) EUROPRENE™ N3380. Torimex Group (Konstantynów Łódzki, Poland): carbon black (N330), naphthenic mineral oil.

### 2.2. Preparation of Rubber Mixes

Two different unfilled elastomer compounds were prepared for testing, representing common vulcanization systems but differing in their type: conventional (CV) and efficient (EV), as well as one carbon black (CB)-filled compound having a semi-efficient vulcanization system (SEV, accelerator-to-sulfur ratio equal to 1.67). The filled NBR sample represents a rubber compound used in the production of gaskets, requiring a balanced participation of particular crosslinks in rubber vulcanizates.

Butadiene-acrylonitrile rubber (NBR) was used as the polymer matrix. The crosslinking agent was elemental sulfur (S_8_), and the vulcanization activator was zinc oxide (ZnO) and stearic acid (ST), which additionally played the role of dispersing agent. A mixture of two accelerators was used in the rubber compounds, one from the thiazole group (primary) and one from the thiuram group (secondary): mercaptobenzothiazole (MBT) and tetramethylthiuram disulfide (TMTD). In the case of the filled compound, carbon black (N330) was used as a filler, and naphthenic mineral oil was applied as a plasticizer. The compositions of the individual rubber compounds are presented in Table 1.

The ingredients were mixed in a Brabender Plasticorder laboratory internal mixer (Brabender, Duisburg, Germany) with a mixing chamber capacity of approximately 60 cm^3^ (fill factor of 0.8) at a temperature of 60 °C. The mixing process began with the plasticization of the rubber for approximately 2 min at 40 rpm. In the case of the filled compound, carbon black, together with plasticizing oil, was incorporated in two portions over the next 5 min (60 rpm) and homogenized for the next 2 min at 80 rpm. Subsequently, stearic acid and zinc oxide were added in two portions over approximately 2 min, with the mixer speed reduced to 30 rpm. Following this, sulfur and accelerators were added separately to the mixture, also in two portions, over an additional period of approximately 2 min at 20 rpm. Finally, the mixture remained in the mixer for homogenization, which was completed after approximately 1 min at 30 rpm. After mixing, the compounds were left to stabilize for 24 h at room temperature. Afterwards, compounds were milled on a David Bridge laboratory two-roll mill (David Bridge & Co., Ltd., Rochdale, UK), equipped with two water-cooled rollers (30 cm long and 15 cm in diameter), rotating at speeds of 18 rpm and 20 rpm, respectively (with a friction ratio of 1.1).

### 2.3. Kinetics of Vulcanization and Vulcanization Process

**Figure 2:**
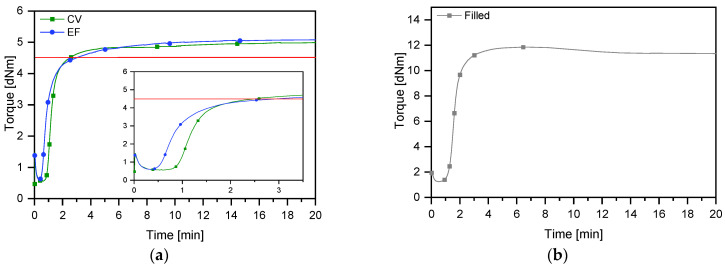
Kinetics of vulcanization for rubber compounds: (**a**) unfilled compounds, (**b**) filled compound.

To study the kinetics of the vulcanization process, samples were cut from the sheeted rubber mixtures and placed in an MDR 2000 rheometer (Alpha Technologies, Akron, OH, USA) operating at an oscillation frequency of 1.667 Hz (100 cycles per minute/cpm) and an oscillation angle of 3° (ISO 6502-2) [39]. The test was carried out at 160 °C for 30 min. The analysis enabled the determination of vulcanization parameters such as torque increase, time to reach a specific torque value, and optimal vulcanization time. Curing curves are presented in Figure 2.

To obtain a comparable crosslink density of compounds CV and EV, the vulcanization time was fixed at a time that achieved a torque of 4.5 dNm (crosspoint on Figure 2). The curing data are presented in Table 2. In the case of the filled compound, vulcanization was performed at t_90_. Rubber samples were prepared in steel molds using a laboratory press at a temperature of 160 °C and a pressure of 20 MPa.

### 2.4. Extraction

Prior to all further analyses, the rubber vulcanizates were purified from low-molecular-weight compounds (unbound elastomer fraction, accelerator residues, free sulfur, etc.) by extraction in boiling acetone using a Soxhlet apparatus according to the ASTM D6814 [40]. The prolonged time from 16 ± 1 h (normatively) to 24 h is to ensure that the extraction is fully completed.

### 2.5. Crosslink Density Analysis by Equilibrium Swelling (Flory–Rehner Equation)

The crosslink density of the rubber vulcanizates was determined by applying the equilibrium swelling method, with calculations based on the Flory–Rehner Equation (1) [41]. For the experiment, five irregularly shaped rubber pieces weighing approximately 50 mg were cut from each sample and placed in sealed weighing bottles containing toluene for 48 h, at room temperature. After this period, the swollen samples were washed with diethyl ether, blotted to remove excess solvent, and weighed. The samples were then placed in empty vessels and dried in an oven for 48 h at 60 °C. After drying, they were weighed again. The relative standard deviation (RSD) during the measurements of crosslink density does not exceed 5%.

(1) $$\nu =-\frac{\ln\left(1-V_{r}\right)+V_{r}+\chi V_{r}^{2}}{V_{0}\left(V_{r}^{\frac{1}{3}}-\frac{2V_{r}}{f}\right)},$$

where ν—crosslink density per unit volume (mol/cm^3^); V_r_—volume fraction of rubber in a swollen sample [-]; V_0_—solvent molar volume (for toluene: V_0_ = 106.9 cm^3^/mol); f—functionality of crosslinks (f = 4, assuming the formation of tetra-functional crosslinks); χ—Flory–Huggins rubber–solvent interaction parameter (0.425 for sulfur crosslinked NBR-toluene pair [42]).

### 2.6. Thiol–Amine Analysis

Thiol-amine analysis was performed to determine the structure of chemical crosslinks in the three-dimensional network of the vulcanizates both before and after exposure to a tetra-*n*-butylammonium fluoride (TBAF) solution, to assess its effect and the resulting changes in the chemical crosslinks. Two solutions were prepared for thiol–amine analysis: a “soft” solution composed of propane-2-thiol (0.4 M) and piperidine (0.4 M) in toluene, and a “hard” solution composed of dodecane-1-thiol (1 M) in piperidine. Rubber pieces of the extracted vulcanizates weighing approximately 250 mg were analyzed. The treatment using the “soft” solution lasted 3 h, whereas the treatment with the “hard” solution was performed for 24 h. All reactions were carried out in sealed glass vessels, under a nitrogen atmosphere, at room temperature. After completion of the analyses, the swollen samples were washed several times with toluene to remove any residual thiol–amine reagents. The samples were then subjected to equilibrium swelling in toluene. The recorded masses after swelling and drying were used to calculate the residual crosslink density in accordance with the methodology described in the preceding section. However, due to the lack of an initial mass value (*m*_0_), the value of (m_s_)* was calculated as:

(2) $$\left(m_{s}^{*})=m_{s}\cdot [1-\;\frac{m_{mineral}}{m_{c}}\right]$$

where *m_s_*—the weight of the dried sample (after swelling) [g]; *m_mineral_*—the content of mineral parts in the rubber mixture [phr]; *m_c_*—the total content of ingredients in the rubber mixture [phr].

The difference between the total crosslink density and that obtained after “soft” thiol–amine analysis corresponds to the proportion of polysulfidic crosslinks in the network. The difference between the crosslink densities obtained after “soft” and “hard” thiol–amine analyses corresponds to the proportion of disulfidic crosslinks. The crosslink density remaining after “hard” (OTAT) thiol-amine analysis represents the fraction of monosulfidic and carbon–carbon crosslinks in the network structure. The relative standard deviation (RSD) during the measurements of crosslink density after analysis does not exceed 10%.

### 2.7. Chemical Devulcanization Using Tetra-n-Butylammonium Fluoride (TBAF) Solutions

To examine the effect of tetra-*n*-butylammonium fluoride on the three-dimensional network structure of the vulcanizates, analyses were conducted using TBAF solutions. TBAF solutions were prepared in toluene or tetrahydrofuran (THF) with variations in concentration, and several analysis conditions, involving different temperatures and reaction times, were tested. The maximum concentration of TBAF in toluene was 0.2 M with full solubility, while in THF a solution of 0.5 M was prepared for comparison. Detailed data are presented in Table 3.

For each analysis, a piece of the rubber sample after extraction (weighing approximately 100–200 mg) was treated with the appropriate solution. All analyses were performed in sealed vessels, under a nitrogen atmosphere, at the specified temperature and reaction time. After the analysis was completed, the samples were washed several times with toluene to remove residual TBAF, and then allowed for equilibrium swelling in toluene to determine the crosslink density after the reactions. The masses recorded after swelling and drying were used to calculate the residual crosslink density, according to the methodology described in Section 2.5 and Section 2.6.

### 2.8. Model Reactions of TBAF with Alkyl Sulfides

To investigate the potential interactions between tetra-*n*-butylammonium fluoride (TBAF) and sulfidic bonds in the three-dimensional network of the crosslinked elastomer, model reactions were carried out using low-molecular dipropyl sulfides: (i) dipropyl monosulfide, (ii) dipropyl disulfide, and (iii) dipropyl trisulfide. For this purpose, tetra-*n*-butylammonium fluoride (TBAF) trihydrate and the corresponding dipropyl sulfide were mixed in a molar ratio of 1:5. The concentration of TBAF in dry THF was 0.13 M, while that of the sulfide was 0.67 M. The reactions were carried out under ambient conditions for 2 h. After that, the excess solvent was removed on a rotary evaporator under reduced pressure (40 °C, 250 mbar). To elucidate the reaction pathway, the crude reaction mixture was analyzed by ^1^H NMR spectroscopy using a JEOL JNM-ECZL 400 MHz spectrometer (JEOL, Tokyo, Japan). Measurements were performed at room temperature in deuterated chloroform (CDCl_3_).

## 3. Results and Discussions

### 3.1. Determination of Crosslink Density and Network Structure of Vulcanizates

#### 3.1.1. Conventional Vulcanizates (CVs)

**Figure 3:**
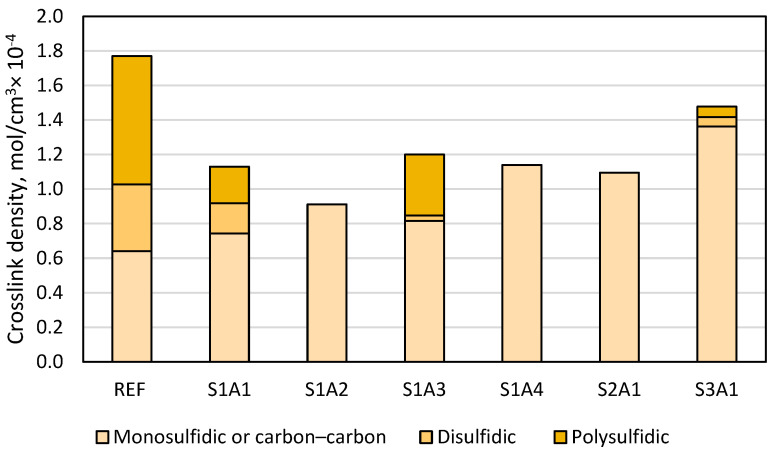
Crosslink density and structure of NBR vulcanizates cured with the conventional sulfur system, before (REF) and after treatment with TBAF solutions (Table 3).

Crosslink density and structure of NBR vulcanizates cured with the CV system, before and after treatment with TBAF solutions (see Table 3), are shown in Figure 3.

The exact values of network density before and after treatment with TBAF solutions, together with the percentage decrease in crosslink density, are presented in Table 4 and Table 5, which show the percentage content of various sulfidic and carbon–carbon bonds.

Subsequent thiol–amine analysis of TBAF-treated rubber vulcanizates cured with the CV sulfur system revealed that most TBAF treatments resulted in significant rearrangement of sulfidic bonds in the crosslinks, although not in a fully selective manner. In some cases (S_1_A_1_, S_1_A_3_, S_3_A_1_), TBAF substantially reduced the ratio of polysulfidic and disulfidic bonds in the network. In other cases (S_1_A_2_, S_1_A_4_, S_2_A_1_), these bonds appeared to be almost entirely converted to monosulfidic or carbon–carbon linkages, as evidenced by thiol-amine analysis. Because no established analytical method currently allows differentiation between monosulfidic and carbon–carbon bonds within the crosslink network, it remains uncertain whether complete desulfurization occurred and the network relies solely on C-C bonds or whether longer sulfur bridges were converted to shorter monosulfidic links. Determination of the total sulfur bound to the polymer matrix could help clarify this issue.

The strongest rearrangement effects were noted at elevated temperatures in toluene solution. Two hypotheses of action can be proposed. First, the nucleophilic fluoride anion may induce partial desulfurization of longer polysulfidic bridges and form a fluoropolysulfide anion of FS_x_^−^ type [34,36], leading to their rearrangement into monosulfidic bonds—thus decreasing the average bond length in a crosslink and reducing the total crosslink density. Second, complete removal of sulfur from crosslinks to fluoropolysulfide anion of FS_x_^−^ type could yield unsaturated carbon–carbon bond formation, which subsequently participates in thiol-ene type “click” reactions forming new C–S linkages [43] and restoring network connectivity through short crosslinks.

An interesting case was observed for sample S_1_A_3_ (25 °C; 24 h of analysis) where crosslink density decreased by about 32%, accompanied by a relatively low proportion of disulfidic and a high proportion of polysulfidic bonds. This behavior may indicate that during the prolonged time of analysis, concurrent formation of new monosulfidic or carbon–carbon bonds resulting from rearrangement of disulfidic crosslinks and reformation of polysulfidic bonds can take place.

#### 3.1.2. Effective Vulcanizates (EFs)

**Figure 4:**
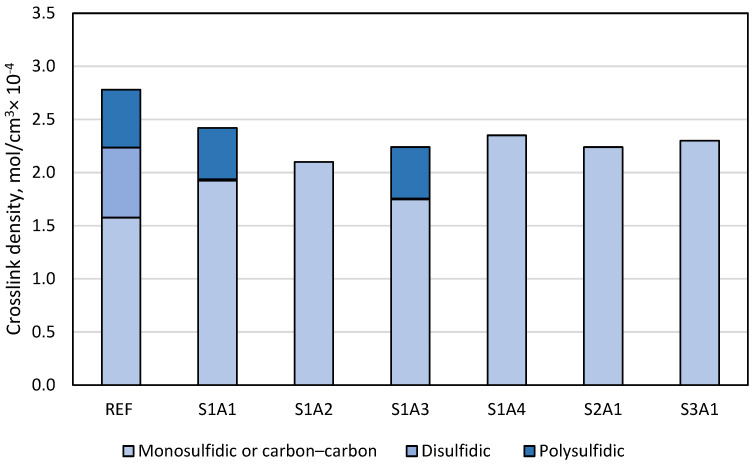
Crosslink density and structure of NBR vulcanizates cured with an effective sulfur system, before (REF) and after treatment with TBAF solutions (Table 3).

The total crosslink density and the ratio of sulfidic and carbon–carbon bonds for rubber vulcanizates cured with an effective sulfur system are shown in Figure 4.

The exact values of network density before and after treatment with TBAF solutions, together with the percentage decrease in crosslink density, are presented in Table 6 and Table 7, which show the percentage content of sulfidic and carbon–carbon bonds in the remaining crosslinks.

NBR vulcanizates crosslinked with the EV sulfur system showed a significantly smaller decrease in crosslink density after TBAF treatment in comparison to rubber vulcanizates crosslinked with the CV sulfur system, which can be attributed to their lower initial content of di- and polysulfidic crosslinks, toward which the fluoride anion is more reactive.

In the case of analysis A1 (3 h) and A3 (24 h) using a 0.2 M solution of TBAF in toluene, at 25 °C, it was observed that the proportion of polysulfidic bonds remained essentially unchanged, while disulfidic bonds were almost completely cleaved. This finding supports the hypothesis that TBAF induces rearrangement of disulfidic bonds into shorter ones even more effectively than of polysulfidic ones.

#### 3.1.3. Filled NBR Vulcanizates

The total crosslink density and structure for filled rubber samples before and after treatment with TBAF solutions (0.2 M in toluene) for 3 h, at 55 °C, are presented in Table 8 and Table 9.

Immersion of the filled rubber vulcanizate in the TBAF solution allows for significant network breakdown of about 48%, which is nearly the same as for unfilled NBR with higher initial polysulfidic bond content in the crosslink structure. This indicates that the chemical devulcanization using TBAF solutions can also be effective in the case of filled rubbers, whose technical significance is of much higher importance.

### 3.2. Investigation of TBAF Reactivity Toward Model Alkyl Sulfides (NMR Study)

**Figure 5:**
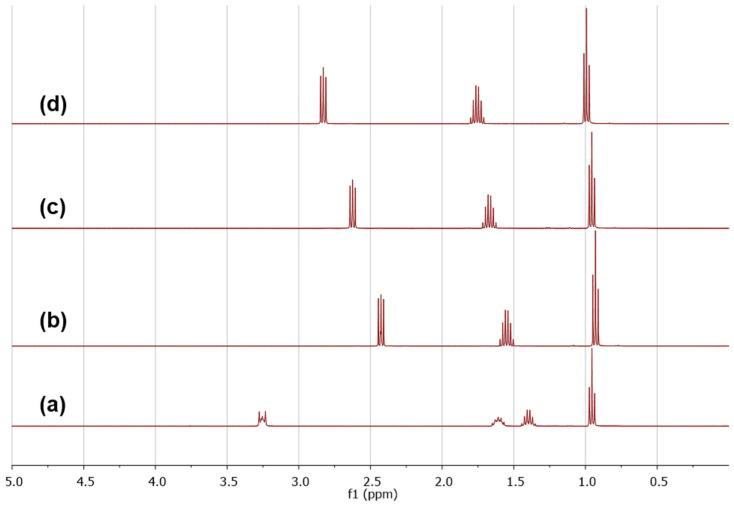
^1^H NMR spectra in the aliphatic region for the starting materials: (**a**) TBAF (tetra-*n*-butylammonium fluoride trihydrate); (**b**) dipropyl monosulfide; (**c**) dipropyl disulfide; (**d**) dipropyl trisulfide.

**Figure 6:**
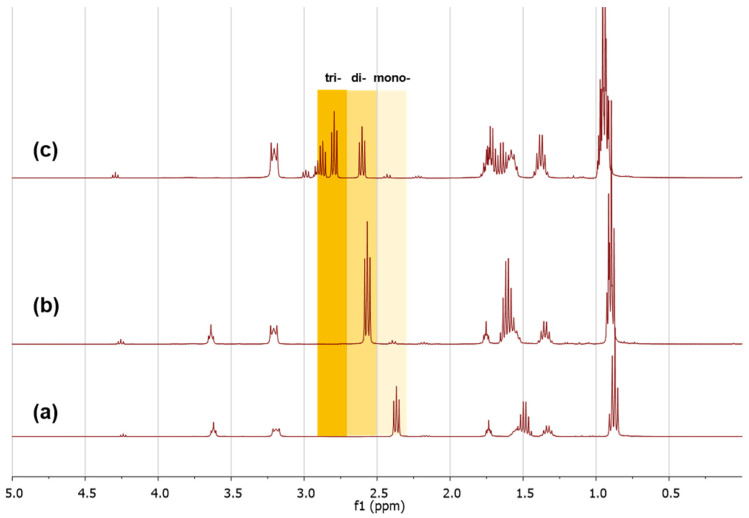
^1^H NMR spectra in the aliphatic region for the model reactions after 2 h: (**a**) reaction mixture of TBAF with dipropyl monosulfide; (**b**) reaction mixture of TBAF with dipropyl disulfide; (**c**) reaction mixture of TBAF with dipropyl trisulfide.

To investigate the potential interactions between TBAF and sulfidic crosslinks in elastomer networks, model reactions were performed using dipropyl mono-, di-, and trisulfides as simplified analogs of rubber crosslinks. ^1^H NMR spectra of the starting materials in the aliphatic region are shown in Figure 5, while spectra of the crude reaction samples after 2 h (a time period similar to that of the rubber analysis) of treatment with TBAF are presented in Figure 6.

A characteristic signal in the ^1^H NMR spectra of dipropyl sulfides appears in the chemical shift range between 2 and 3 ppm corresponding to the methylene protons (–CH_2_–) directly attached to the sulfur atoms (dipropyl monosulfide: δ 2.41–2.45, dipropyl disulfide: δ 2.61–2.64; dipropyl trisulfide: δ 2.81–2.83). These signals are well-resolved and do not overlap with the characteristic resonances of TBAF, enabling clear monitoring of the reactions.

As shown in Figure 6c, dipropyl trisulfide undergoes significant conversion to dipropyl disulfide within the reaction time, with a minor amount of dipropyl monosulfide also formed (ratio tri-:di-:mono- 1:0.79:0.04). In contrast, dipropyl disulfide exhibits only very slight conversion to dipropyl monosulfide (Figure 6b) (ratio di-:mono—1:0.04). Importantly, dipropyl monosulfide shows no detectable conversion under the same conditions (Figure 6a); for example, no formation of hex-3-ene, which would result from complete desulfurization of the monosulfide, was observed. One possible reason is that TBAF is unable to cleave monosulfidic C–S bonds under the applied conditions. These model reactions are in good agreement with the crosslink density analyses performed directly on the samples of rubber vulcanizates and support the hypothesis that TBAF selectively cleaves longer polysulfidic and, to a lesser extent, disulfidic linkages, while leaving monosulfidic and carbon–carbon crosslinks intact.

## 4. Conclusions

This study demonstrates that tetrabutylammonium fluoride (TBAF) is an interesting devulcanization aid, being able to cleave polysulfidic and disulfidic crosslinks in sulfur-vulcanized NBR. The degree of network degradation was strongly dependent on the initial crosslink structure, with CV sulfur-cured rubber containing high proportions of longer sulfidic bonds showing reductions in crosslink density of up to 49%. In comparison, EF sulfur-cured rubber exhibited more modest reductions (up to 24%). However, in the case of filled rubber compounds, whose technical significance is of much higher importance, the chemical devulcanization of rubber using TBAF solution allowed for significant network breakdown (48%). The most effective treatment conditions were 0.2 M TBAF in toluene, at 55 °C, for 3 h.

Model compound ^1^H NMR spectroscopy studies using dipropyl sulfides confirmed that TBAF acts as a reducing agent toward polysulfidic and disulfidic bonds, while leaving monosulfidic crosslinks intact. Polysulfidic bonds were extensively cleaved, whereas disulfidic ones, despite their higher bond dissociation energies, were substantially, and even more preferably, degraded.

The observed reduction in crosslink density of sulfur vulcanizates of NBR approaching nearly 50% under mild conditions suggests that TBAF has potential as a chemical devulcanization aid for diene rubbers. However, further studies are needed to evaluate its effectiveness and optimization under industrially relevant thermomechanical conditions for assessing its practical applicability for rubber recycling.

## Figures and Tables

**Table 1:** Composition of the rubber samples studied.

Ingredient	CV (phr ^1^)	EV (phr)	Filled (phr)
NBR	100	100	100
Carbon black	–	–	60
Naphthenic oil			15
Zinc Oxide	3	3	3
Stearic Acid	1	1	2
TMTD	0.56	2.25	1.5
MBT	0.44	1.75	1
Sulfur	1.5	0.6	1.5
(MBT + TMTD)/Sulfur ratio	0.67	6.67	1.67

^1^ Parts per hundred rubber.

**Table 2:** Selected rheometric results for rubber compounds.

Parameter	CV	EV	Filled
M_max_ ^1^, dNm	5.0	5.1	11.9
M_90_ ^2^, dNm	4.5	4.6	10.7
t_90_ ^3^, min	2.6	3.5	2.5
t(4.5 dNm) ^4^, min	2.6	3.1	–

^1^ Maximum torque, ^2^ 90% of maximum torque, ^3^ Optimum vulcanization time, ^4^ Time corresponding to a torque of 4.5 dNm.

**Table 3:** Conditions of analysis using TBAF solutions.

Sample	TBAF Concentration (M)	Solvent	Temperature (°C)	Analysis Time (h)
S_1_A_1_	0.2	Toluene	25	3
S_1_A_2_	0.2	Toluene	55	3
S_1_A_3_	0.2	Toluene	25	24
S_1_A_4_	0.2	Toluene	55	6
S_2_A_1_	0.2	THF	25	3
S_3_A_1_	0.5	THF	25	3

S—solution, A—analysis.

**Table 4:** Total crosslink density of NBR vulcanizates cured with CV sulfur system before (REF) and after treatment with TBAF solutions.

Sample	Network Density (mol/cm^3^, ×10^−4^)	Percentage Decrease in Crosslink Density, %
REF	1.77	-
S_1_A_1_	1.13	36
S_1_A_2_	0.91	49
S_1_A_3_	1.20	32
S_1_A_4_	1.14	35
S_2_A_1_	1.10	38
S_3_A_1_	1.48	17

S—solution, A—analysis.

**Table 5:** Percentage content of sulfidic and carbon–carbon bonds in the crosslinks of NBR vulcanizates cured with CV sulfur system.

Sample	Percentage Content, %		
	Monosulfidic and Carbon–Carbon	Disulfidic	Polysulfidic
REF	36	22	42
S_1_A_1_	66	15	19
S_1_A_2_	100	0	0
S_1_A_3_	68	3	29
S_1_A_4_	100	0	0
S_2_A_1_	100	0	0
S_3_A_1_	92	4	4

S—solution, A—analysis.

**Table 6:** Total crosslink density of NBR vulcanizates cured with EF sulfur system before (REF) and after treatment with TBAF solutions.

Sample	Network Density (mol/cm^3^, ×10^−4^)	Percentage Decrease in Crosslink Density, %
REF	2.87	-
S_1_A_1_	2.42	13
S_1_A_2_	2.10	25
S_1_A_3_	2.24	20
S_1_A_4_	2.35	16
S_2_A_1_	2.24	20
S_3_A_1_	2.30	17

S—solution, A—analysis.

**Table 7:** Percentage content of sulfidic and carbon–carbon bonds in the crosslinks of NBR vulcanizates cured with EF sulfur system.

Sample	Percentage Content, %		
	Monosulfidic and Carbon–Carbon	Disulfidic	Polysulfidic
REF	57	23	20
S_1_A_1_	79	1	20
S_1_A_2_	100	0	0
S_1_A_3_	78	0	22
S_1_A_4_	100	0	0
S_2_A_1_	100	0	0
S_3_A_1_	100	0	0

S—solution, A—analysis.

**Table 8:** Total crosslink density of CB-filled NBR vulcanizates before (REF) before and after treatment with TBAF solutions.

Sample	Network Density (mol/cm^3^, ×10^−4^)	Percentage Decrease in Crosslink Density, %
REF	4.34	-
S_1_A_2_	2.28	48

**Table 9:** Percentage content of sulfidic and carbon–carbon bonds in the crosslinks of CB-filled NBR vulcanizates.

Sample	Percentage Content, %		
	Monosulfidic and Carbon–Carbon	Disulfidic	Polysulfidic
REF	36	19	45
S_1_A_2_	92	0	8

## Data Availability

The original contributions presented in this study are included in the article. Further inquiries can be directed to the corresponding author.

## Abbreviations

The following abbreviations are used in this manuscript:

TBAF	Tetra-*n*-butylammonium fluoride
S	Solution
A	Analysis
